# Fluid management strategies and their interaction with mechanical ventilation: from experimental studies to clinical practice

**DOI:** 10.1186/s40635-023-00526-2

**Published:** 2023-07-21

**Authors:** Eduardo Butturini de Carvalho, Denise Battaglini, Chiara Robba, Manu L. N. G. Malbrain, Paolo Pelosi, Patricia Rieken Macedo Rocco, Pedro Leme Silva

**Affiliations:** 1 Laboratory of Pulmonary Investigation, Carlos Chagas Filho Biophysics Institute, Federal University of Rio de Janeiro, Rio de Janeiro, Brazil; 2 University of Vassouras, Rio de Janeiro, Brazil; 3 IRCCS Ospedale Policlinico San Martino, Genoa, Italy; 4 Department of Surgical Sciences and Integrated Diagnostics (DISC), University of Genoa, Genoa, Italy; 5 First Department of Anesthesiology and Intensive Therapy, Medical University of Lublin, Lublin, Poland; 6 International Fluid Academy, Lovenjoel, Belgium

**Keywords:** Acute respiratory distress syndrome, Liberal fluids, Restrictive fluids, Fluid management, Mechanical ventilation, Hemodynamics

## Abstract

Patients on mechanical ventilation may receive intravenous fluids via restrictive or liberal fluid management. A clear and objective differentiation between restrictive and liberal fluid management strategies is lacking in the literature. The liberal approach has been described as involving fluid rates ranging from 1.2 to 12 times higher than the restrictive approach. A restrictive fluid management may lead to hypoperfusion and distal organ damage, and a liberal fluid strategy may result in endothelial shear stress and glycocalyx damage, cardiovascular complications, lung edema, and distal organ dysfunction. The association between fluid and mechanical ventilation strategies and how they interact toward ventilator-induced lung injury (VILI) could potentiate the damage. For instance, the combination of a liberal fluids and pressure-support ventilation, but not pressure control ventilation, may lead to further lung damage in experimental models of acute lung injury. Moreover, under liberal fluid management, the application of high positive end-expiratory pressure (PEEP) or an abrupt decrease in PEEP yielded higher endothelial cell damage in the lungs. Nevertheless, the translational aspects of these findings are scarce. The aim of this narrative review is to provide better understanding of the interaction between different fluid and ventilation strategies and how these interactions may affect lung and distal organs. The weaning phase of mechanical ventilation and the deresuscitation phase are not explored in this review.

**Supplementary Information:**

The online version contains supplementary material available at 10.1186/s40635-023-00526-2.

## Take-home message

Ventilatory management may be affected by restrictive and liberal fluid strategies due to physiological interaction between heart–lung, possibly yielding to distal organ damage in critical ill patients. Pre-clinical studies evaluated the effects of different fluid strategies on ventilator-induced lung injury during assisted ventilation, at different PEEP levels, as well as after an abrupt decrease in PEEP.

**Supplementary Information:**

The online version contains supplementary material available at 10.1186/s40635-023-00526-2.

## Background

Mechanical ventilation (MV) often results in impaired gas exchange, hemodynamic instability, and injury to endothelial cells. Intravenous (IV) fluid therapy is often required in patients undergoing MV to restore hemodynamics and distal organ perfusion [1, 2]. According to Paracelsus (1493–1541) and previous authors [1]: “*Dosis sola facit venenum*”, all things are poison and it is the dose that makes something poisonous. Optimizing tissue perfusion and oxygen delivery while preventing fluid overload is a challenge in critically ill patients. Restrictive fluid management [1, 3] can be associated with peripheral hypoperfusion and distal organ damage [4–7]. However, a more liberal approach could increase mortality because it may lead to endothelial cell damage, lung and peripheral tissue edema, increased intra-abdominal pressure, and gastrointestinal and renal dysfunction [5, 6, 8–17]. Notwithstanding, the myriad of modes and settings for MV, such as positive end-expiratory pressure (PEEP) and tidal volume (*V*_t_), can have distinct impacts on cardiovascular physiology, as well as volemic status and fluid balance [7, 18, 19]. Variations in pleural (*P*_pl_) and transpulmonary (*P*_tp_) pressures caused by assisted or controlled MV have been shown to affect the preload and afterload as well as capillary transvascular filtration pressures [20–23]. Some experiments have shown that mismatch between fluid and ventilatory strategies can worsen ventilator-induced lung injury (VILI) as well as reduce cardiac output and tissue perfusion [7, 18, 22]. In specific scenarios, such as acute respiratory distress syndrome (ARDS), more than 60% of patients are dependent on inotropic drugs to achieve an adequate arterial pressure [24] and often require IV fluids as part of hemodynamic support. Although protective MV and a restrictive fluid strategy have been suggested for critically ill patients, this combination may affect distal organs [25, 26]. However, evidence evaluating the interaction between fluid therapies with different modes of MV is scarce. Most clinical studies investigating the impact of restrictive and liberal fluid therapies on organ damage and mortality do not provide detailed information concerning the MV strategy or vice versa.

This narrative review aims to provide better understanding of the crosstalk between fluids and MV strategies and the impact of this interaction on lung and distal organs. The weaning phase of MV and the deresuscitation phase are not explored in this review.

### Physiologic rationale: heart–lung interactions and distal organ damage

Because of its location, the heart is inevitably subjected to the mechanical forces of the lungs, namely, *P*_pl_ and *P*_tp_ [27–29]. These forces can have an impact on at least two factors regulating cardiac output: venous return and the heart’s ability to deal with preload during the systolic phase [28, 29]. During spontaneous breathing, *P*_pl_ is negative during the expiratory phase and even more negative during inspiration [30], favoring systemic venous return in normo- or hypervolemia. During positive pressure ventilation, the increase in intrathoracic pressure increases right atrial pressure, reducing systemic venous return [27, 29, 31, 32]. The left ventricle, in turn, has its afterload reduced by a lower transmural pressure and a transiently increased preload by a higher alveolar pressure that squeezes blood toward the left ventricle [29]. Left ventricular afterload is reduced due to an increase in pleural pressure during MV, whereas left ventricular transmural pressure tends to decrease because it is the difference between ventricular and pleural pressure. Thus, during MV when P_pl_ is positive, transmural pressure decreases. However, over time, the transmural pressure may recover due to increased stressed volume or vessel tone, which in turn can increase the mean systemic filling pressure, favoring venous return [33]. Under protective MV, about 70% ± 27% of airway pressure (*P*_aw_) is transmitted to juxtacardiac pleura, 37% ± 17% to the pericardium, and 43% ± 11% to the vena cava; these numbers can be even higher when chest wall compliance is reduced [32]. Organ perfusion pressure is determined by the difference between inflow and outflow pressure, therefore a higher intrathoracic pressure during positive pressure ventilation may potentially compromise organ perfusion, ultimately leading to organ damage. Because the right ventricle has less contractile reserve than the left ventricle, intrathoracic pressure and afterload swings during the respiratory cycle have a greater effect on the former than on the latter [34]. This concept becomes especially important in ARDS, where hypoxic vasoconstriction can increase right ventricular afterload, which may lead to right cardiac failure [35].

**Fig. 1:**
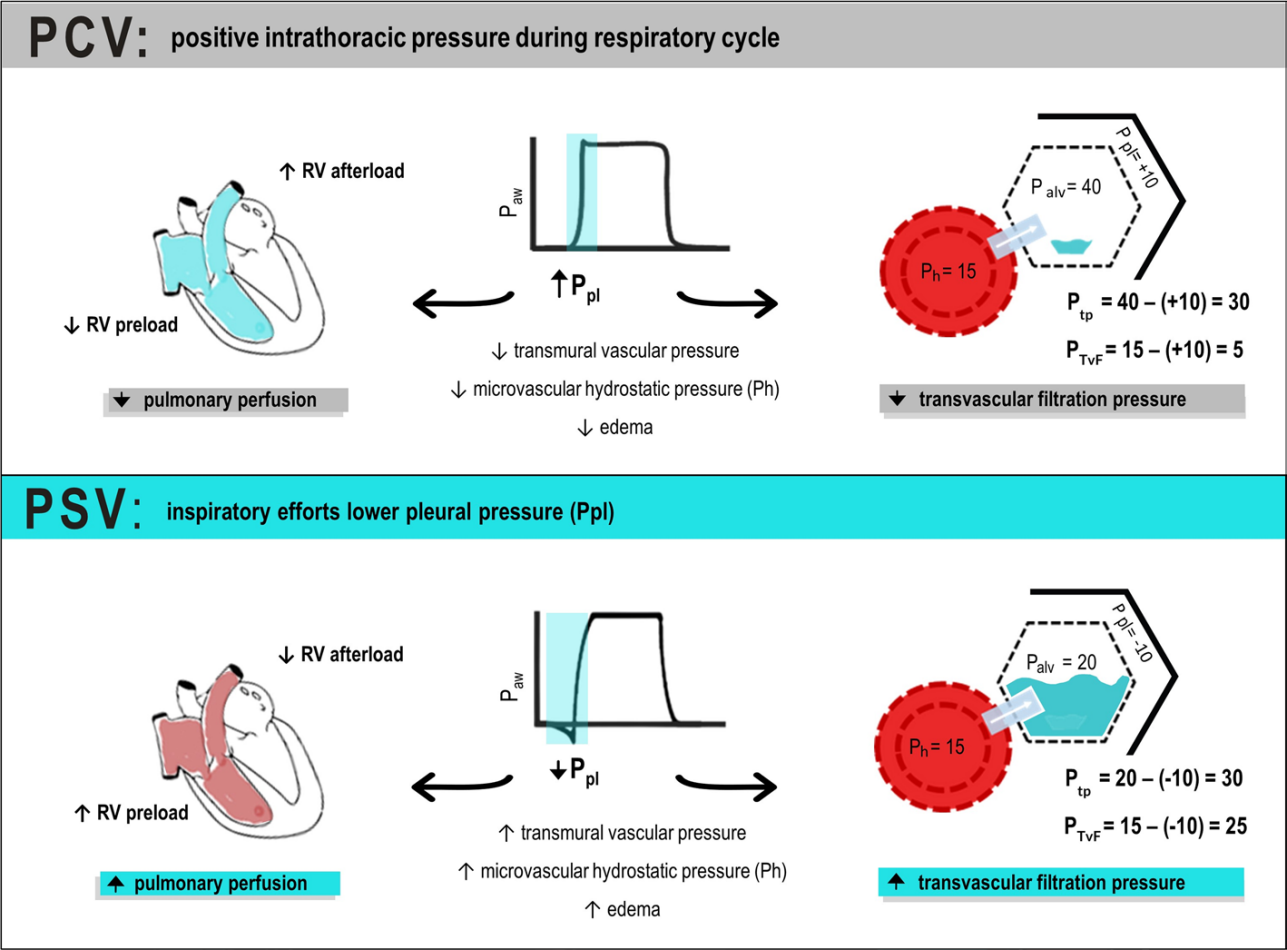
Hemodynamic changes in controlled and assisted mechanical ventilation. In pressure-support ventilation, pleural pressure (*P*_pl_) is lowered by inspiratory efforts, leading to higher venous return and lower right ventricular (RV) afterload. Increased transmural pressure (caused by the decrease in *P*_pl_ from inspiratory effort) increases hydrostatic pressure (*P*_h_) in the microvasculature, worsening edema. Increased flow in lung vessels may also lead to shear stress, causing further endothelial damage and protein and fluid leak into alveolar space. Transvascular filtration pressure (*P*_TvF_) is higher in pressure-support ventilation than in pressure control ventilation (even at the same transpulmonary pressure (*P*_tp_) given by the difference between alveolar pressure (*P*_alv_) and *P*_pl_). Adapted from Vieillard-Baron et al. [20]

Pulmonary transvascular filtration pressure is defined as the difference between vascular hydrostatic pressure (*P*_h_) and *P*_pl_. In spontaneous breathing and assisted ventilation (such as pressure-support ventilation [PSV]), a more negative inspiratory *P*_pl_ may increase transvascular filtration pressure. In the presence of extremely negative *P*_pl_, due to intense inspiratory effort against obstructed airways, a sudden increase in pulmonary transvascular filtration pressure and lung edema may occur [36]. The association between negative *P*_pl_, resulting from spontaneous breathing (or assisted ventilation), and hypervolemia (that may be caused by liberal fluids) increases *P*_h_, thus increasing the risk of edema, which can be even worse in the presence of increased vascular permeability [37]. These mechanisms are presented in Fig. 1.

The MV mode, whether spontaneous or controlled, can change intrathoracic pressures and may lead to changes in hemodynamics [35]. Given the significant hemodynamic impact of the heart–lung interactions in critically ill patients, the use of hemodynamic tests and indices have been widely endorsed to better predict volume responsiveness [38].

### Liberal and restrictive fluid management: search for an objective definition

Restrictive versus liberal fluid management have been compared in various settings, albeit not clearly defined [5, 7] due to the different terminologies adopted in clinical studies. “Conservative” [7, 39–44] and “restrictive” [34, 45–50] are used interchangeably without any clearly defined pattern regarding fluid rates. There is an overall lack of consensus on this; for example, a “restrictive” approach (6 ml/kg/h) has been compared with a “conservative” approach (12 ml/kg/h) [51]. Higher fluid rates are frequently named “liberal”, and older clinical studies use labels such as “standard”, “high volume”, and even “aggressive” [11, 52–54]. Experimental and clinical studies have so far used the term “liberal” over a remarkably wide range from 1.2 to 12 times the fluid rates referred to as restrictive (Additional file 1: Table S1) [10, 45, 46, 48, 55–57].

### Impact of restrictive versus liberal fluid management on lung and distal organ damage

Recent surgical and intensive care guidelines—such as Enhanced Recovery from Anesthesia and Surgery (ERAS) and UK guidelines for the management of ARDS—support restrictive fluid therapies [6, 58]. Evidence points to a significant association between liberal fluids, hypervolemia, and glycocalyx damage (shown by increased plasma syndecan-1 [59–62], hyaluronic acid [60], and heparan sulfate [17, 61]). Also, increased central venous and capillary hydrostatic pressures may reduce organ perfusion pressure and facilitate lung interstitial edema [2]. In murine models of acute lung injury, increased capillary hydrostatic pressure caused by liberal fluids was shown to promote perivascular lung edema than a restrictive approach [7, 18]. In addition, higher fluid rates could even increase the risk of developing ARDS after surgery [63]. Thus, the main consequences of liberal fluids may be lung edema, reduced oxygen delivery, and distal organ damage.

Organ damage may also be caused by insufficient fluid therapy. For example, an excessively restrictive approach can lead to renal hypoperfusion and further functional impairment [6, 34, 64]. The Surviving Sepsis Campaign indicates that there is not enough evidence to recommend restrictive fluids in the first 24 h of resuscitation in patients with signs of hypoperfusion and volume depletion [4]. The BaSICS study showed no difference in 90-day mortality in patients in the intensive care unit (ICU) when comparing slower versus faster crystalloid infusion rates [65]. In major abdominal surgery, restrictive fluids resulted in higher acute kidney injury, need for renal replacement therapy, and surgical-site infection rates than a liberal approach (8.6% versus 5.0%, 16.5% versus 13.6%, and 0.3% versus 0.9%, respectively; all *p* < 0.05) [34].

Even in specific syndromes, such as ARDS, it seems that distinct phenotypes (hyper- or hypoinflammatory) may respond differently to restrictive or liberal approaches, as demonstrated in the cohort in the Fluids and Catheters Treatment Trial [39]. In this study, subphenotype I (mainly trauma, aspiration, or pneumonia) had lower 90-day mortality under restrictive fluid management (26% versus 18%), whereas patients with subphenotype II (sepsis as a primary risk factor and a lower central venous pressure) had lower mortality under a liberal fluid management (40 versus 50%). Thus, fluid therapy should be individualized according to the patient’s specific needs. ERAS guidelines strongly recommend avoiding excessively restrictive or liberal fluid regimes during lung surgery [66]. ERAS also supports goal-directed fluid therapy with dynamic monitoring over a liberal fluid management for renal transplantation [67]. There is no mention of the relationship between fluid and MV strategies.

Over the last decades, attempts to improve outcomes by fluid balance have ranged from dehydration and negative fluid balance to normovolemia and even moderate hypervolemia as primary therapeutic goals [68]. The 2018 European Society of Intensive Care Medicine consensus statement on fluid therapy in neurointensive care [69] suggests targeting normovolemia during fluid replacement in patients with a brain injury. It also suggests fluid balance, arterial blood pressure, and variables such as cardiac output and blood lactate as primary and safety endpoints to titrate fluids.

### Impact of assisted versus controlled mechanical ventilation on lung and distal organ damage

Some studies have suggested that assisted spontaneous breathing modes such as PSV could be associated with a reduction in VILI and length of stay in the ICU, and an increase in ventilator-free days in experimental and clinical studies [70–75]. Although assisted ventilation can prevent the harmful effects of controlled MV, intense inspiratory efforts during assisted ventilation can also dramatically change the intrathoracic pressures. This can lead to increased lung perfusion and transvascular filtration pressures and facilitate alveolar edema. Increased inspiratory efforts may lead to patient self-inflicted lung injury (P-SILI) and negative pressure edema [76]. This situation could be even worse in lungs with endothelial injury. A recent study [77] hypothesized that intrapulmonary dyssynchrony (i.e., *pendelluft*, defined in this study as the percentage of the *V*_t_ that moves during inspiration from the non-dependent to the dependent lung region) could be a leading mechanism for VILI and P-SILI. The authors showed that regional *pendelluft* during BiPAP may reflect local swings in *P*_pl_ during spontaneous breathing and be associated with an increase in specific inflammatory biomarkers in patients with ARDS. On the other hand, muscle paralysis and controlled ventilation were shown to be safer than spontaneous breathing in severe acute lung injury in an animal model [78].

Protective controlled ventilation (low *V*_t_ and moderate-to-high PEEP after recruitment maneuver) is associated with a lower incidence of acute kidney injury [79, 80] and reduced pulmonary complications and mortality [6, 66]. However, depending on airway pressures, it affects hemodynamics [22]. Higher PEEP and peak inspiratory pressures may be associated with distal organ damage as long as hemodynamics are altered and vasopressin secretion is increased [18, 81, 82]. Also, a high PEEP may produce a masking effect on the PaO_2_/FiO_2_ ratio due to changes in hemodynamics—namely a reduction in cardiac output and a proportional reduction in venous admixture.

### Interaction between mechanical ventilation and fluid management

In 1947, researchers first showed a reduction in renal blood flow, glomerular filtration rate, and urine output during positive airway pressure [83]. Since then, only a few studies have assessed the interaction between fluid and ventilatory strategies. Here, we discuss the evidence comparing lung and organ damage under assisted or controlled ventilation in restrictive and liberal fluid management.

PSV is a frequently used mode of assisted ventilation in patients who are breathing spontaneously. Intense inspiratory efforts during assisted ventilation could lead to hemodynamic impairment [7, 20, 21, 70], higher transpulmonary pressures [78], increased lung perfusion, and likely P-SILI. Judicious adjustment of delta pressure during PSV [84] or assisted modes and higher PEEP levels [85, 86] can help prevent P-SILI and possibly protect patients during assisted ventilation, mainly with liberal fluid management. The increased transvascular pressure (caused by increased inspiratory efforts and liberal fluids) might cause vascular shear stress, ongoing endothelial damage, and alveolar edema in patients with high capillary permeability, as observed in sepsis and ARDS [17, 24]. The combination of liberal fluids and PSV increased alveolar diffuse damage and MMP-9 gene expression and decreased specific biomarkers associated with epithelial integrity (occludin, *zona occludens*-1, and claudin-4) [7]. Although no differences in kidney morphology were observed, NGAL (neutrophil gelatinase associated lipocalin) expression during PSV was lower with a liberal fluids approach compared with a restrictive fluids approach.

The effects of controlled ventilation on cardiac output and tissue perfusion partially depend on *V*_t_. In this context, both pressure control ventilation (PCV) and volume control ventilation (VCV) with the same tidal volume resulted in comparable cardiac output during MV. However, PCV may result in higher cardiac outputs when lower *V*_t_ are used [35, 87, 88]. The reduction in cardiac output observed in VCV partially explains the negative impact of positive pressure ventilation on renal function. However, other mechanisms may play a role in the development of kidney injury, including redistribution of intrarenal blood flow, hyperactivation of the sympathetic nervous system, and the action of inflammatory mediators [89]. In an attempt to improve cardiac output, liberal fluids strategy may be advised. First, to improve cardiac output, the patient must be fluid responsive (if cardiac output response is negligible, fluid should be stopped) [38, 90]; second, stretched alveolar epithelial cells can have disrupted tight junctions [23]; in this case, a high hydrostatic pressure could worsen lung edema. It has been demonstrated that in VCV, *V*_t_ is positively and linearly correlated with *P*_pl_. Vascular filtration pressure for an intrathoracic vessel is the difference between hydrostatic vascular pressure and *P*_pl_, and researchers have shown that superior vena cava transmural pressure decreased during inspiration in VCV, whereas right atrium transmural pressure did not [32]. This reduction of transmural pressure in intrathoracic vessels could be protective with a liberal fluid management, because it would reduce transvascular filtration and formation of edema. Although no clinical studies have investigated this interaction, chloride-rich fluids can promote renal vasoconstriction, which could be even worse in the presence of positive pressure ventilation [90].

It has been shown that PSV in combination with a restrictive fluid strategy resulted in less lung epithelial damage in a model of acute lung injury. One likely explanation is that damage to tight junctions, which was identified by a decrease in occludin expression, was observed in animals during PSV combined only with a liberal fluid strategy but not with a restrictive fluid approach. The interaction between the mode of MV and the fluid strategy may have a mechanistic relationship [7]. In addition, edema may increase further if tight junction connections, which are constitutive in epithelial and endothelial structural cells, are lost during the stretch movements produced by tensile stress in PSV.

The choice of PEEP levels should also take into account the volume status. It has been shown in clinical studies that a high PEEP level can decrease kidney function despite the fluid strategy because it can increase peak inspiratory pressures [18]. The combination of high PEEP and liberal fluids worsened lung injury in a murine model of ARDS [18]. In addition, an abrupt decrease in PEEP has been shown to increase club cell-16 protein, a marker of alveolar epithelial cell damage marker, in an experimental model of ARDS. When combined with a liberal fluid management, it worsened diffuse alveolar damage and increased the levels of inflammatory and endothelial cell damage biomarkers [19]. Table 1 summarizes the main findings from pre-clinical studies investigating interactions between MV and fluid management.

**Table 1:** Main findings from pre-clinical studies on mechanical ventilation and fluid management interactions

Rocha et al. [19]	Animal model: Lung injured (intratracheal *E. coli* LPS) male Wistar rats randomized to receive restrictive (10 mL/kg/h) or liberal (30 mL/kg/h) fluids and mechanical ventilation under protective VT (6 ml/mg) and an abrupt or gradual PEEP decrease (directly from 9 to 3 cmH_2_O or the same decrease in 30 min)
	Main findings: • Liberal fluids were associated with higher right and left ventricular end-diastolic areas in echocardiographic measurements despite PEEP decrease rate • PAT/PET ratio was higher in abrupt than in gradual PEEP decrease despite fluid management • Combined liberal fluids and abrupt PEEP decrease yielded more diffuse alveolar damage and higher interleukin-6 and endothelial growth factor expression, • Restrictive fluids and gradual PEEP decrease yielded higher zona occludens-1 expression, suggesting epithelial cell preservation • Abrupt PEEP decrease group showed higher club-16 protein expression regardless of fluid management, suggesting higher alveolar epithelial cell damage • Kidney injury markers were higher in liberal fluid management despite PEEP decrease strategy
Carvalho et al. [7]	Animal model: Lung injured (intratracheal *E. coli* LPS) male Wistar rats randomized to receive restrictive (minimum fluids to keep MAP ≥ 70 mmHg) or liberal (~ 4 times fluids received by restrictive groups) and protective PCV or PSV ventilation
	Main findings: • In PSV groups, restrictive fluids led to reduced diffuse alveolar damage and lung edema, preservation of occludin and claudin-4 and higher expression of zona occludens-1 in lungs (suggesting tight junctions’ integrity) • Liberal fluids groups reduced interleukin-6 and neutrophil gelatinase-associated lipocalin expression, regardless of ventilatory strategy
Felix et al*.* [18]	Animal model: Lung injured (intratracheal *E. coli* LPS) male Wistar rats randomized to receive restrictive (5 ml/kg/h) or liberal (40 ml/kg/h) fluids and volume-controlled ventilation under protective *V*_T_ (6 ml/kg)
	Main findings: • Liberal fluids led to a higher transpulmonary plateau pressure than restrictive fluids • A combination of high PEEP (9 cmH_2_O) and liberal fluids led to higher inflammatory gene expression than low PEEP-liberal fluids and high PEEP-restrictive fluids • Fluid management did not affect lung mechanical power and heterogeneity index between high and low PEEP groups • Liberal fluids led to higher perivascular edema despite PEEP strategy • Under liberal fluids, high PEEP was associated with more intense epithelial and extracellular matrix damage • Acute kidney injury biomarkers were higher in high PEEP regardless of fluid management

*PAT/PET* pulmonary acceleration time to pulmonary ejection time ratio, *PEEP* positive end-expiratory pressure, *PCV* pressure-controlled ventilation, *PSV* pressure-support ventilation, *V*_*T*_ tidal volume

### Clinical implications

The clinical evidence is scarce and mainly limited to a few experimental studies, therefore the effects of interaction between MV and fluid management on organ damage are still poorly understood. Thus, it would be reckless to address clinical recommendations based on it. Nevertheless, some possible clinical implications from these experimental studies should be pointed out. First, whenever restrictive or liberal fluids are strongly recommended, caution should be taken when choosing the ventilatory strategy. Especially in the early phase of resuscitation, when large amounts of fluids are warranted, assisted ventilation and intense inspiratory efforts may cause higher transvascular filtration pressures, vascular edema, and epithelial cell damage, especially with concurrent lung and endothelial damage. Experimental data show that despite protective ventilation, high and rapid intravenous fluid boluses can be associated with worsened lung injury and respiratory function [91]. In this setting, careful titration of fluid therapy or opting for controlled ventilation is probably beneficial. In contrast, whenever a patient receives a combined strategy with restrictive fluids and controlled MV, distal organ damage should be closely monitored, especially when high PEEP is used or when decreased lung compliance leads to high peak inspiratory pressures. Ventilatory settings should also be carefully titrated because the association between higher PEEPs and liberal fluids may worsen lung injury, and the association between higher PEEPs and restrictive fluids may aggravate distal organ damage.

## Conclusions

The understanding of physiological heart–lung interaction is fundamental to optimize fluid strategies and mechanical ventilation setting. Accepted definitions of restrictive or liberal fluid strategies do not exist. Both restrictive and liberal fluid strategies may lead to hypoperfusion and edema of distal organs, respectively. Assisted ventilation may cause self-inflicted lung injury associated with liberal fluid strategies, while controlled ventilation may impair hemodynamics, and thus distal organ damage with restrictive fluid strategies, especially when high PEEP levels are used. Gradual transitioning of ventilatory patterns is suggested to promote lung protection due to the impact on vascular compartment. Optimization of the type and mechanical ventilation setting should consider careful titration of fluid strategies in critically ill patients.

## Supplementary Information

**Additional file 1: Table S1.** Restrictive and liberal fluid strategies in experimental and clinical studies.

## Data Availability

Not applicable.

## Abbreviations

ARDS: Acute respiratory distress syndrome
BiPAP: Biphasic positive airway pressure
CPAP: Continuous positive airway pressure
ERAS: Enhanced recovery from anesthesia and surgery
ICU: Intensive care unit
MV: Mechanical ventilation
NGAL: Neutrophil gelatinase associated lipocalin
PCV: Pressure control ventilation
PEEP: Positive end-expiratory pressure
P-SILI: Patient self-inflicted lung injury
PSV: Pressure support ventilation
VCV: Volume-controlled ventilation
VILI: Ventilator-induced lung injury
