# Supplementary material for: Fluid management strategies and their interaction with mechanical ventilation: from experimental studies to clinical practice
Source: Intensive Care Med Exp. 2023 Jul 21;11:44. doi: 10.1186/s40635-023-00526-2 (PMC10359242; doi:10.1186/s40635-023-00526-2)
Supplement: Supplementary file 1 — Additional file 1: Table S1. Restrictive and liberal fluid strategies in experimental and clinical studies. [file 40635_2023_526_MOESM1_ESM.docx]

**Table S1** Restrictive and liberal fluid strategies in experimental and clinical studies

| Study | No. | Species | | Fluid rates | | |
| --- | --- | --- | --- | --- | --- | --- |
|  |  |  |  | Restrictive | | Liberal |
| **Experimental studies** | | | | | | |
| Marjanovic et al., 2009 [55] | 21 | R | | 3 ml/kg/h | | 36 ml/kg/h |
| Herbert et al., 2016 [43] | 12 | M | | No fluids | | 100 μl/h |
| Ingelse et al., 2016 [47] | 32 | R | | 2.5 ml/kg/h | | 6.5 ml/kg/h |
| Rocha et al., 2021 [19] | 35 | R | | 10 ml/kg/h | | 30 ml/kg/h |
| **Clinical studies** | | | | | | |
| Holte et al., 2004 [48] | 48 | | 15 ml/kg/h | | 40 ml/kg/h | |
| Nisanevich et al., 2005 [47] | 152 | | 4 ml/kg/h IO | | 10 ml/kg B + 12 ml/kg/h IO | |
| Wiedemann et al., 2006 [10] | 1000 | | 4231±120 ml | | 5030 ± 133 ml | |
| Holte et al., 2007 [45] | 20 | | 12.5 ml/kg B + 15 ml/kg/h IO | | 12.5 ml/kg B + 40 ml/kg/h IO | |
| Holte et al., 2007 [46] | 48 | | 5 ml/kg/h | | 10 ml/kg/h | |
| Futier et al.,2010 [51] | 70 | | 6 ml/kg/h | | 12 ml/kg/h | |
| Lobo et al., 2011 [48] | 88 | | 4 ml/kg/h | | 12 ml/kg/h | |
| Grant et al., 2016 [49] | 330 | | 6 ml/kg/h | | 12 ml/kg/h | |
| Kotlińska-Hasiec et al., 2017 [56] | 72 | | 10 ml/kg/h IO + 3 ml/kg/h PO | | 30 ml/kg/h IO + 5 ml/kg/h PO | |
| Shin et al., 2018 [57] | 92,094 | | ≤900 ml | | >2700 ml | |
| Myles et al., 2018 [34] | 3000 | | 6.5 ml/kg/h | | 10.9 ml/kg/h | |
| Alimian et al., 2020 [50] | 72 | | 746 ± 127 ml | | 1824 ± 310 ml | |
| Bihari et al., 2021 [91] | 161 | | 5 ml/kg B + 5 ml/kg/h IO + 0.8 ml/kg/h PO | | 10 ml/kg B + 8 ml/kg/h IO + 1.5 ml/kg/h PO | |
| Schol et al., 2021 [52] | 252 | | 0.75–1× blood losses | | 1.5–2× blood losses | |
| Shapiro et al., 2023 [53] | 1563 | | 1267 ml/24 h | | 3400 ml/24 h | |

B: bolus; IO: intra-operative; M: mice; PO: post-operative; R: rats.

*Some studies presented data as total infused volume. In these cases, the infusion rate was calculated by dividing this rate by the average body weight of the population.

**Whenever the duration of fluid therapy was more than 1 day, only data for the first day were presented.

91. Ingelse SA, Juschten J, Maas MAW, Matute-Bello G, Juffermans NP, van Woensel JBM, et al. (2109) Fluid restriction reduces pulmonary edema in a model of acute lung injury in mechanically ventilated rats. PLoS One 14(1):e0210172. https://doi.org/10.1371/journal.pone.0210172

92. Holte K, Klarskov B, Christensen DS, Lund C, Nielsen KG, Bie P, et al. (2004) Liberal versus restrictive fluid administration to improve recovery after laparoscopic cholecystectomy: a randomized, double-blind study. Ann Surg 240(5):892. <https://doi.org/10.1097/01.sla.0000143269.96649.3b>

93. Grant FM, Brennan MF, Allen PJ, DeMatteo R, Kingham TP, D’Angelica M, et al. (2016) Prospective randomized controlled trial of liberal versus restricted perioperative fluid management in patients undergoing pancreatectomy. Ann Surg 264(4):591. <https://doi.org/10.1097/SLA.0000000000001846>

94. Alimian M, Mohseni M, Moghadam OM, Siamdoust SAS, Moazzami J. (2020) Effects of liberal versus restrictive fluid therapy on renal function indices in laparoscopic bariatric surgery. Anesthesiol Pain Med 10(5):e95378. <https://doi.org/10.5812/aapm.95378>

95. Schol PBB, de Lange NM, Woiski MD, Langenveld J, Smits LJM, Wassen MM, et al. (2021) Restrictive versus liberal fluid resuscitation strategy, influence on blood loss and hemostatic parameters in mild obstetric hemorrhage: an open-label randomized controlled trial (REFILL study). PLoS One 16(6):e0253765. https://doi.org/10.1371/journal.pone.0253765

96. Shapiro NI, Douglas IS, Brower RG, Brown SM, Exline MC, Ginde AA, et al. (2023) Early restrictive or liberal fluid management for sepsis-induced hypotension. N Engl J Med. https://doi.org/10.1056/nejmoa2212663
